# Cost-Effectiveness of Dolutegravir in HIV-1 Treatment-Experienced (TE) Patients in France

**DOI:** 10.1371/journal.pone.0145885

**Published:** 2015-12-29

**Authors:** Gilles Pialoux, Anne-Geneviève Marcelin, Nicolas Despiégel, Caroline Espinas, Hélène Cawston, Laurent Finkielsztejn, Audrey Laurisse, Céline Aubin

**Affiliations:** 1 Service des Maladies Infectieuses et Tropicales, AP-HP Hôpital Tenon, Paris, France; 2 Service de Virologie, AP-HP, Hôpital Pitié-Salpêtrière, INSERM-Sorbonne Universités, UPMC Univ Paris 06, UMR_S 1136, Paris, France; 3 Optum, Paris, France; 4 Mapi Group (previously Optum), Paris, France; 5 ViiV, Marly-le-Roi, France; 6 GSK, Marly-le-Roi, France; University of Pittsburgh Center for Vaccine Research, UNITED STATES

## Abstract

**Objectives:**

To evaluate the cost-effectiveness of a new generation integrase inhibitor (INI), dolutegravir (DTG), in France, in treatment-experienced (TE) and INI-naïve HIV-infected adults with at least two classes resistance compared to raltegravir (RAL), by adapting previously published Anti-Retroviral Analysis by Monte Carlo Individual Simulation (ARAMIS) model.

**Methods:**

ARAMIS is a microsimulation Markov model with a lifetime time horizon and a monthly cycle length. Health states are defined as with or without opportunistic infection and death. In the initial cohort, efficacy and safety data were derived from a phase III study comparing DTG to RAL. Antiretroviral treatment algorithms, accounting for patient history, were based on French guidelines and experts opinion. Costs are mainly including treatment costs, routine HIV and opportunistic infection care, and death. Utilities depend on CD4+ cell count and the occurrence of opportunistic infections.

**Results:**

The ARAMIS model indicates in the TE population that DTG compared to RAL over a life time is associated with 0.35 additional quality-adjusted life years (QALY; 10.75 versus 10.41) and additional costs of €7,266 (€390,001 versus €382,735). DTG increased costs are mainly related to a 9.1-month increase in life expectancy for DTG compared with RAL, and consequently a longer time spent on ART. The incremental cost-effectiveness ratio (ICER) for DTG compared with RAL is €21,048 per QALY gained. About 83% and 14% of total lifetime costs are associated with antiretroviral therapy and routine HIV care respectively. Univariate deterministic sensitivity analyses demonstrate the robustness of the model.

**Conclusion:**

DTG is cost-effective in the management of TE INI naive patients in France, from a collective perspective. These results could be explained by the superior efficacy of DTG in this population and its higher genetic barrier to resistance compared to RAL. These data need to be confirmed with longer-term real life data.

## Introduction

Human immunodeficiency virus (HIV) is a retrovirus that infects immune cells, resulting in a progressive decline in CD4+ cell count and immune function, and consequently leaving patients susceptible to acquired immunodeficiency syndrome (AIDS) with increased risk of being affected by life-threatening opportunistic infections (OI) and cancer.

Due to substantial improvements in the clinical management of HIV and the use of highly active antiretroviral therapy (HAART) in the last 15 years, the life expectancy of HIV-infected individuals has increased from approximately eight years from time of diagnosis and presentation to over 30 years.[1, 2]

HIV also engenders a large economic burden for the individual, health care system and society. In Europe, mean annual costs of care for an HIV/AIDS patient have been estimated to range from €9,894 (in 2007 in Italy) to €20,170 (in 2010 in France), dependent upon the country and the proportion of patients being treated with antiretroviral therapy (ART).[3, 4] In France, the economic cost of HIV was modelled in a study by Sloan et al. (2012), which reported that the estimated lifetime cost of treating an HIV-infected person was €535,000/patient (€320,700 [discounted]), assuming a mean life expectancy of 27.4 years.[4]

French guidelines for treatment experienced HIV patients state that the choice of treatment should be based on treatment history in terms of adherence, tolerance and resistance profile. In case of virological failure, a combination of three active molecules, including a PI/r, is recommended.[5] These combinations have been shown to have transformed HIV infection from a fatal disease into a chronic condition.[6] In 2010, 118,450 patients are treated with ARTs and covered by the French Healthcare Insurance System. However, despite advancements, 16% of those experienced virological failure.[7]

The Plato project, which follows several European cohorts including patients who failed to three different ART classes, showed that the introduction of new treatment classes with new mechanisms of action led to an improvement in viral load suppression and lower cross-resistance.[8] Dolutegravir (DTG—Tivicay^®^) is a new generation integrase inhibitor (INI) that has recently been approved by the European Medicines Agency (granted date– 20^th^ of January 2014) to be used in combination with other ARTs to treat HIV-infected adults and adolescents (≥12 years of age) in both treatment-naïve and treatment-experienced (TE) patients.[9] DTG has a high plasma half-life in patients, supporting once-daily dosing without pharmacokinetic boosters and has demonstrated promising efficacy results in patients with multiclass resistance.[10]

The objective of this study is to evaluate the cost-effectiveness of dolutegravir (DTG) versus raltegravir (RAL) in treatment-experienced, INI-naïve adult patients with virological failure in France. The model was adapted following French guidelines on “Choices in Methods for Economic Evaluation”.[11]

## Methods

### Model structure

**Fig 1:**
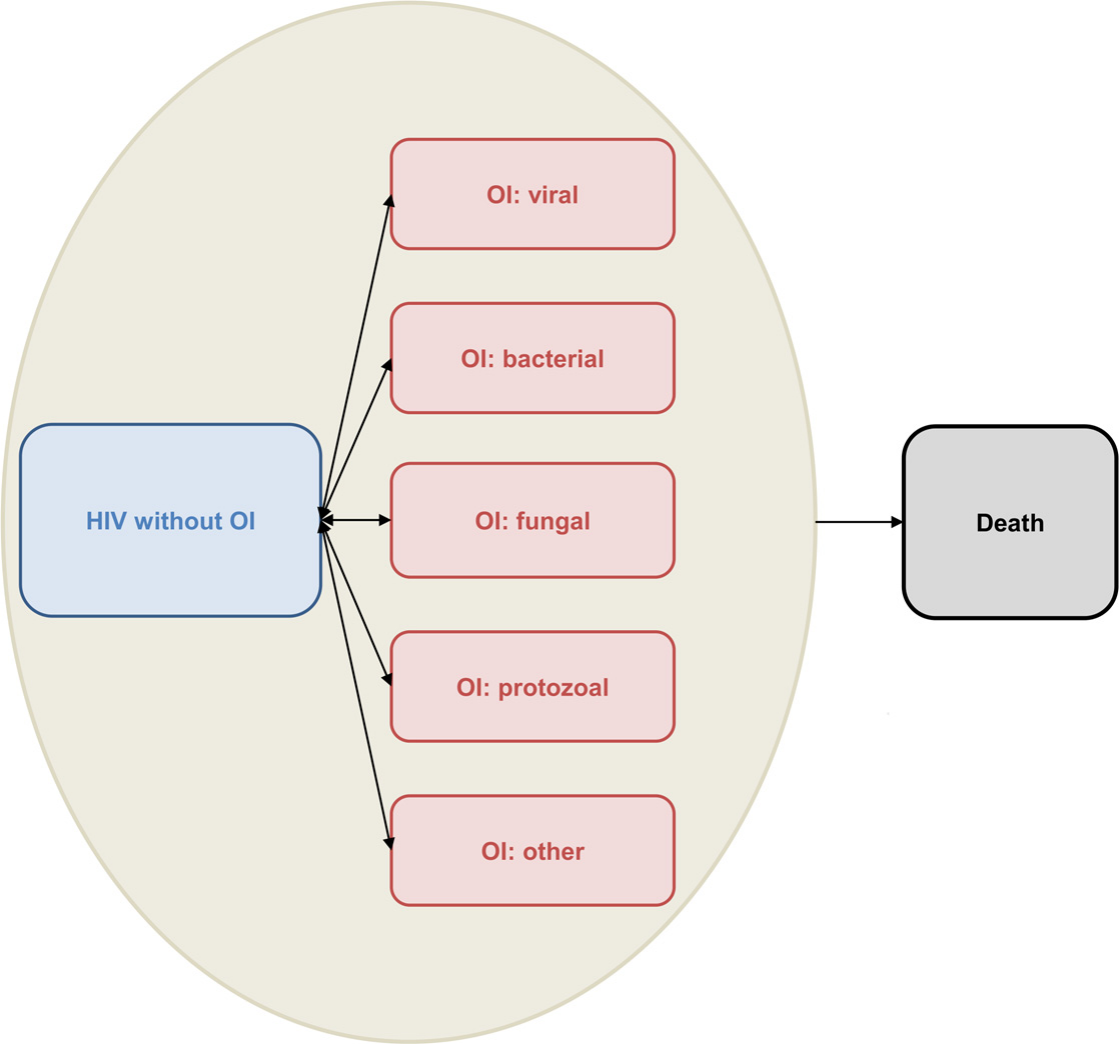
Model structure. HIV: Human Immuodeficiency Virus; OI: Opportunistic Infection.

The Anti-Retroviral Analysis by Monte Carlo Individual Simulation (ARAMIS)-DTG model is a Markov model adapted from the reknown CEPAC (Cost-effectiveness of Preventing AIDS Complications) model on the prevention of complications of AIDS.[12] This micro-simulation model has been adapted to predict the lifetime costs and health outcomes of TE HIV-infected patients treated with DTG or RAL. Individual patients transit through mutually exclusive health states (with or without OI and death) with continually adjusted probabilities of disease progression based on their characteristics and disease history (Fig 1). Microsimulation models are particularly adapted to modelling HIV disease, as it allows modelling complex relationships such as viral load, CD4+ cell count, opportunistic infections, and keeps track of the starting characteristic of each individual along with the occurrence of clinical events. One month cycle length is considered.

**Fig 2:**
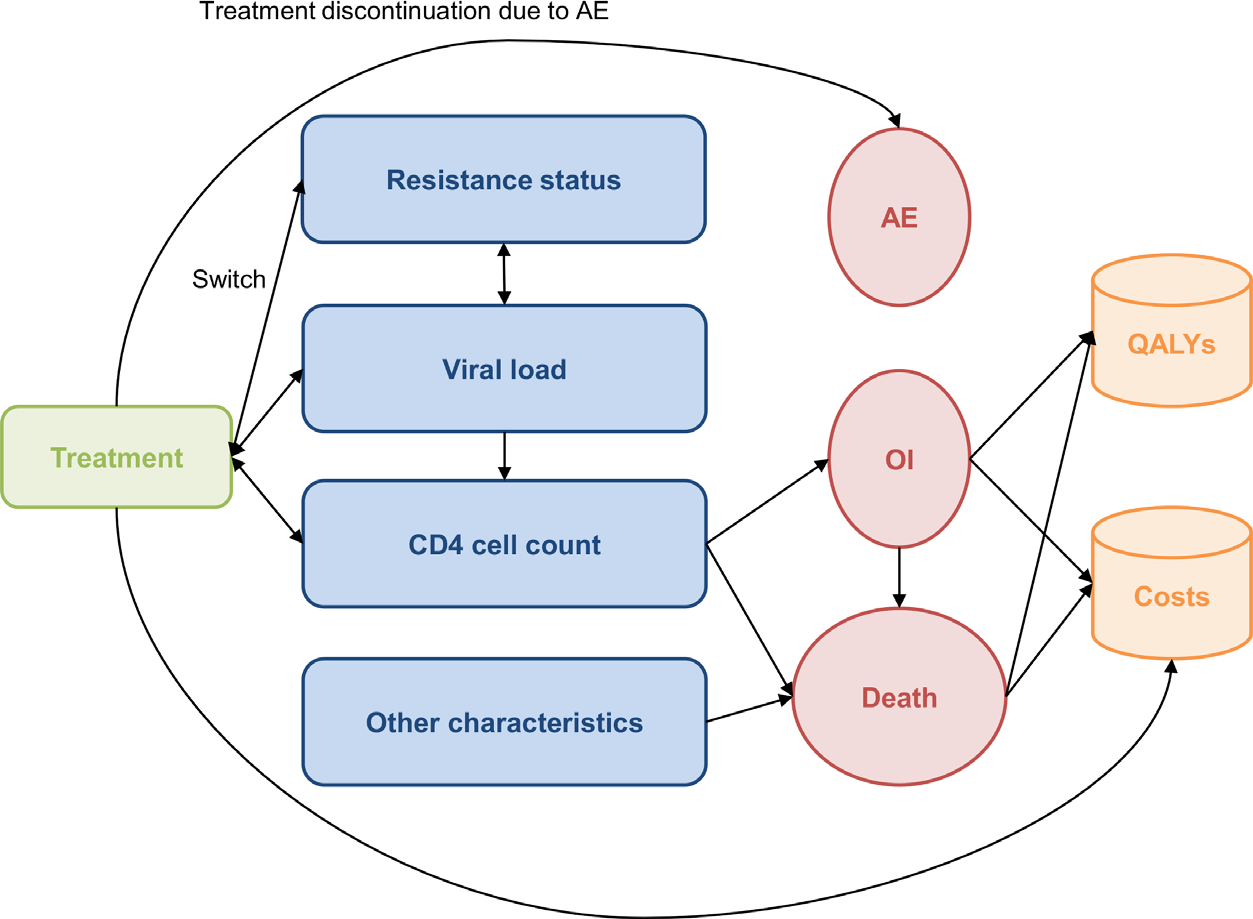
Influence diagram. AE: Adverse event; HIV: Human Immunodeficiency Virus; OI: Opportunistic Infection; QALYs: Quality Adjusted Life Years.

Patients can experience natural progression of HIV infection including change in CD4 cell count, opportunistic infections (OI, lasting three months), treatment failure, switching to alternative treatments and death. Each patient is treated over his lifetime by successive lines of therapy based on the occurrence of virologic failure, opportunistic infections (OI) and/or adverse events. Successive treatment lines depend on the treatment history and resistance. Fig 2 describes the interactions between the components of the model such as the treatment, patient characteristics (age, gender, viral load, CD4+ cell count), events (OIs, adverse events, deaths), and outcomes (costs and quality adjusted life years [QALYs]).

Individuals can experience virologic failure (virologic failure in the first year of treatment and virologic rebound from the second year), changes to their CD4 cell count, OIs, adverse events (acute or long-term toxicity), or can change line of treatment. These changes are determined by the individual characteristics of patients, HIV resistance profile, previous medical history and treatments. All of these changes are dealt within the model by tracking variables. Several individual variables are defined at the patient inclusion in the model (age, gender, viral load, CD4+ cell count, HIV resistance profile), while some of these may be modified over the patient’s lifetime (age, viral load, CD4+ cell count, HIV resistance profile). In particular, a virologic failure with the development of INI resistance after the first model treatment line impacts the characteristics of the subsequent treatments administered (e.g., the treatment types, their efficacy and the possible number of successive effective treatments). Patients may present OIs with probabilities of occurrence depending on their initial characteristics and factors changing over time such as the CD4+ cell count and duration of treatment. Patients with an OI history will have a higher probability to present further OIs and death.[13]

Each health state has an associated cost and utility. Administered treatment lines, the natural progression of the disease and the events associated with HIV determine the costs and number of QALYs accrued during the patients’ lifetime. Following French guidelines on modelling, which are in line with recommended approaches for assessing the cost effectiveness of new interventions in other countries (United Kingdom, Scotland, Canada, Belgium), only direct costs are considered, including costs associated with ART, laboratory tests and treatment switches, routine HIV care, OI management and death. Utilities are dependant on CD4 cell count, and the occurrence of OIs. Costs and QALYs are discounted at 4% up to 30 years with a reduction of up to 2% thereafter, and a collective perspective is considered.[11, 14]

The outcomes for the model are (1) total costs, (2) QALYs, (3) incremental cost-effectiveness ratios (ICERs) and (4) clinical outcomes (occurrence of OI, cause of death, progression to AIDS, AIDS-free survival, treatment history, time on ART, time on successful therapy, and time on failing therapy). The appropriate number of simulations to provide stable and reproducible estimates is estimated to be 500,000. A random number generator set is used for simulations to ensure reproductibility. The model was developed using *Excel* 2007 software for inputs and outputs sheets, and all calculations were performed with Visual Basic for Application.

### Model inputs

**Treatment lines.** Treatment algorithms were defined by an independent scientific committee composed of three experts, according to their clinical practice, French treatment guidelines, and treatment combinations used in clinical trials and reported in the French Named Patient Program (NPP) report.[5, 15] Three or four treatment lines are considered in the model, depending on the INI resistance status (Table 1). Individuals discontinue treatment due to adverse events (AE), treatment failure or death.

**Table 1:** Treatment algorithm considered in HIV patients.

Treatment algorithm				
Treatment line in the model	Resistance status			
**1**	**DTG + OBT**_**1**_		**RAL + OBT**_**1**_	
** **	**No additional resistance**	**INI resistance**	**No additional resistance**	**INI resistance**
**2**	DTG + OBT_2.0_	OBT_2.2_	OBT_2.1_	OBT_2.2_
**3**	Salvage 1	Salvage 2	Salvage 1	Salvage 2
**4**	Salvage 2		Salvage 2	
**Treatment combinations considered within OBTs or salvage therapies**				
**OBT**_**1**_ ^a^ *	DRV/r + TDF (29%); LPV/r + TDF (17%); DRV/r + ETR (15%); LPV/r (15%); ATV/r + TDF (15%); DRV/r + MVC (9%)			
**OBT**_**2.0**_ ^b^ ^,^ ^c^	NRTI + PI/r (30%); NRTI + PI/r+ NNRTI (25%); NRTI + PI/r + MVC (18%); NRTI + PI/r + NNRTI + MVC (15%); NRTI + PI/r + T-20 (12%)			
**OBT**_**2.1**_ ^b^	DRV/r + ETR + TDF (38%); DRV/r + MVC + TDF (19%); DRV/r + T20 + TDF (19%); DRV/r + ETR + MVC + TDF (12%); DRV/r + ETR + T-20 + TDF (12%)			
**OBT**_**2.2**_ **, salvage 1, salvage 2** ^b^ ^,^ ^c^	NRTI + PI/r + NNRTI + MVC (56.25%); NRTI + PI/r + T-20 (43.75%)			

ATV/r: ritonavir-boosted Atazanavir; DRV/r: ritonavir-boosted Darunavir; DTG: Dolutegravir; ETR/r: ritonavir-boosted Etravirine; INI: Integrase Inhibitor; MVC: Maraviroc; NRTI: Nucleoside Reverse Transcriptase Inhibitor; NNRTI: Non-Nucleoside Reverse Transcriptase Inhibitor; OBT: Optimized Background Therapy; PI/r: ritonavir-boosted Protease Inhibitor; RAL: Raltegravir; T-20: enfuvirtide; TDF: Tenofovir

^a^ SAILING[10]

^b^ Expert panel opinion

^c^ NPP report (February 2013)[15]

*Only treatment combinations used in more than 5% in patients included in SAILING were considered for the OBT_1_ definition. Their proportion of utilisation in the model were adjusted to 100% for the OBT_1_ cost calculation.

In the first line of the model, patients receive either DTG or RAL, both in combination with optimized background therapy (OBT_1_). The latter was based on the OBT used in the SAILING study. OBT types given in more than 5% of the cases were taken into consideration. Patient transitioned from the first treatment line to the second one, contingent upon events such as treatment discontinuation or virologic failure. Patients who did not develop any resistance received DTG and OBT_2.0_ or RAL + OBT_2.1_ only if previously treated with DTG or RAL_._ In case of INI resistance, OBT_2.2_ was prescribed. Salvage lines 1 and 2 consisted of the same combination as OBT_2.2_. All OBTs and salvage therapies are described in Table 1.

#### Efficacy parameters

The efficacy inputs used in the model are summarised in Table 2. The three main efficacy parameters considered in ARAMIS are: the virologic efficacy (defined as HIV RNA viral load below 50 copies/mL), the CD4+ cell count gain and the virologic late failure or rebound after initial viral suppression (defined as HIV RNA viral load superior to 50 copies/mL).

**Table 2:** Efficacy data, by treatment line in the model.

	Annual virologic suppression rate (< 50 copies/mL)	Monthly late failure rate (> 50 copies/mL)		Annual CD4+ cell count increase (Cells/µL)		
	Week 0 to 48	Week 48 to 96	Thereafter	Week 0 to 48	Week 48 to 96	There-after
**Line 1**						
DTG + OBT_1_	70.9% [64;78]^a^	0.76%^b^	2.75%^b^	190^a^	14^b^	19^b^
RAL + OBT_1_	63.7% [57;70]^a^	0.76%^b^	2.75%^b^	189^a^	14^b^	19^b^
**Line 2**						
No resistance	60.6% [52;69]^b^	0.76%^b^	2.75%^b^	176^b^	14^b^	19^b^
INI resistance	50.8% [39;63]^b^	0.76%^b^	2.75%^b^	176^b^	14^b^	19^b^
**Salvage lines**						
Salvage 1	10% [2;23]^c^	0.88%^b^ ^,^ ^c^	2.75%^b^ ^,^ ^c^	118^c^	0	0
Salvage 2	7% [1;19]^c^	0.88%^b^ ^,^ ^c^	2.75%^b^ ^,^ ^c^	118^c^	0	0

CD4: Cluster of Differentiation 4; DTG: Dolutegravir; INI: Integrase Inhibitor; mL: milliliter; OBT: Optimized Background Therapy; RAL: Raltegravir; μL: microliter

^a^SAILING[10]

^b^ BENCHMRK[16, 17, 19, 20]

^c^MOTIVATE[12, 18, 21]

For the first year, the immunovirologic responses in TE patients treated with DTG or RAL in combination with OBT_1_, were derived from the pivotal phase III clinical trial (SAILING).[10] Efficacy data were obtained from the BENCHMRK trial in second line, and from the MOTIVATE trial for salvage lines, assuming equal efficacy for both arms.[12, 16–18] Beyond one year, equal efficacy between treatment arms is assumed and for all three efficacy parameters derived from the BENCHMRK and MOTIVATE trials.[12, 19–21]

In the model, successful ART regimen is defined as a viral load below 50 copies/mL after 48 weeks. The CD4+ cell count is assumed to increase each cycle when viral suppression is maintained. For the first 11 months, the CD4+ cell count gain is assumed to be treatment-specific, reflecting data from the SAILING trial.[10] As observed in the TORO and MOTIVATE studies, two thirds of the CD4+ cell count increase observed at 48 weeks are assumed to occur within the first two months,[22, 23] and a slower rate of CD4+ cell count increase is considered between months 3 and 11. Beyond 11 months, the CD4+ cell count gain is modelled equally in both treatment arms for successfully suppressed patients. The CD4+ cell count is limited to a maximum of 1,000 cells/ μL in the model, based on expert opinion. Furthermore, it is assumed that the CD4+ cell count gain obtained in suppressed patients treated with subsequent treatment lines cannot be higher than for those treated with their first ART. In the absence of evidence describing the late failure pattern over time, late failure rates are assumed to be constant between 48 and 96 weeks, and between 96 and 240 weeks. In addition, late failure rates were multiplied by three compared to the published BENCHMRK values, to reflect higher late failure rates reported in observational studies.[20, 24]

#### Costs and resource use

The following direct costs are considered, according to a collective perspective: costs of care performed routinely, costs of the management of acute opportunistic infections, ART costs, costs of laboratory tests (CD4+ cell count, HIV viral load and genotypic resistance testing), initiation and processing costs line change (physician and nursing visits) and costs of death (care associated with the care of patients in the month before their death). Costs associated with HIV routine care (determined by CD4+ cell count), OI management and death were derived from a French study by Sloan *et al*. (2012) (Table 3).[4] Resource use associated with HIV routine care include inpatient hospitalizations, outpatient visits, clinical procedure, prophylactic treatments of opportunistic infections as well as treatment for HBV, HCV, hyperlipidemia and diabetes, and laboratory tests (excluding CD4+ cell count, HIV viral load and genotyping tests).[4] The costs of ART, laboratory tests and treatment switches (nurse and physician visits as well as additional costs of tests) were obtained from the French Healthcare Insurance Database consulted in October 2014 (Table 3).[25–29]

**Table 3:** Monthly costs (€2012–2014).

**ART costs, by regimen **		
DTG/RAL	612	
OBT_1_	986	
OBT_2.0_ wMean (min; max)	1,698 (1,056; 2,740)	
OBT_2.1_ wMean (min; max)	2,218 (1,294; 2,978)	
OBT_2.2_/Salvage wMean (min; max)	2,491 (2,291; 2,740)	
**Rountine HIV care costs** *		
**CD4+ cell count level**	**No OI history**	**OI history**
>500	224	275
351–500	296	398
201–350	377	775
101–200	826	1,071
51–100	887	1,091
0–50	1,224	938
**OI costs, by type** *		
Bacterial	6,518	
Fungal	9,119	
Protozoal	9,608	
Viral	11,873	
Other OI	6,467	
**Costs associated with death** *		
No OI history	7,548	
OI history (>30 days)	14,351	
OI history (≤30 days)	11,985	

ART: AntiRetroviral Therapy; CD4: Cluster of Differentiation 4; DTG: Dolutegravir; HIV: Human immunodeficiency virus; Max: Maximum; Min: Minimum; OBT: Optimized Background Therapy; OI: Opportunistic Infection; RAL: Raltegravir; wMean: Weighted Mean

* Routine HIV care, OI and death costs were inflated to 2012 prices using the health services consumer price index from the National Institute of Statistics and Economic Studies (INSEE)[39]

#### Utilities

Utilities stratified by CD4+ cell count were pooled from EQ-5D scores from DTG phase III studies in treatment-naïve patients: SPRING-2, SINGLE and FLAMINGO.[30–33] Utilities of 0.830, 0.860, 0.870 and 0.900 were applied to patients with CD4+ cell counts ≤50, 51–100, 101–200 and >200/mm^3^, respectively. Utilities associated with OIs were taken from Paltiel *et al*. (1998): 0.561 for bacterial and viral; 0.652 for protozoal and fungal; and 0.561 for other OIs.[34] Disutilities associated with AEs and OIs are not considered in the model.

### Sensitivity analyses

Deterministic sensitivity analyses (DSA) were performed to explore the impact of variation of key input parameters and assumptions on model outcomes.

Univariate analyses focused on the parameters expected to impact model results with the applied ranges defined by estimates of variance (e.g. confidence intervals or min/max for ART costs) or based on clinical expert-validated assumptions. These included the uncertainty surrounding the three efficacy parameters: virologic response at week 48 (using the 95% confidence interval, CI), virologic rebound between week 48 and week 96 and CD4+ cell count increase at week 48 (based on clinical trial values). Sensivity analyses were also performed on the efficacy of subsequent treatment lines using the 95% CI; the costs of subsequent or salvage treatment lines using either the cheapest or the most expensive ART combination for all considered backbones (reflecting the uncertainty on the treatments that are actually prescribed in patients who failed on multiple regimens). Alternative data sources for utilities by CD4+ cell count and disutilities associated with adverse events leading to discontinuation were also considered. They were taken from Kauf *et al*. (2008), which was based on five open label trials of HAART including 1,327 patients who responded to the SF-36 questionnaire.[35]

Structural sensitivity analyses were conducted to evaluate the robustness of the model to some model assumptions—modifying the time horizon (15 years versus lifetime), the OI duration (1 month versus 3 months), the absence of resistance development to DTG and its comparators and the use of alternative assumptions for discounting (0% and 6% annual probability applied to costs and QALYs). In addition, two sensitivity analyses were performed on the CD4+ cell count decline for patients having failed the last model treatment line. For the first one, the CD4+ cell count decline was based on TN-patients data from the multicentre AIDS cohort study (worse case scenario) while no CD4+ cell count was considered for the other (optimistic but unrealistic assumption).[36]

No probabilistic sensitivity analysis (PSA) was carried out as the estimated running time of a PSA using the current model was too long. Given the number of parameters included in the model, the 500 replications required to cover the range of distributions accurately would represent around 10,000 hours of analysis (417 days). When reducing to 140 replications of 50,000 simulations, the uncertainty around the model was clearly amplified with adding uncertainty around the base estimates due to sampling. Therefore, the uncertainty around the model is only presented considering one-way sensitivity analyses.

## Results

### Base case

Base case results, displayed in Table 4, demonstrate that over a lifetime, life expectancy and QALYs are higher in the DTG arm compared to the RAL arm (18.06 vs. 17.30 and 10.75 vs. 10.41, respectively) in TE INI-naive patients. Patients treated with DTG live longer and healthier than those treated with RAL (gain of 4.14 quality-adjusted months). This gain is primarily driven by the superior efficacy of the DTG demonstrated in the SAILING trial, and its higher barrier to resistance which results in a greater lifetime incremental QALY gain for DTG compared to RAL (0.35 QALY). In addition, DTG patients are estimated to stay on first line for approximately 3.6 months longer.

**Table 4:** Base-case results.

	DTG arm	RAL arm	Incremental
**Cost-effectiveness results (€2012–2014)**			
Total costs	€ 390,001	€ 382,735	€ 7,266
Undiscounted Life Years (LYs)	18.06	17.30	0.76
Discounted LYs	12.03	11.65	0.37
QALYs	10.75	10.41	0.35
ICER			€ 21,048
**Mean time spent on treatment: undiscounted, in months (years)**			
First treatment line	39.7 (3.3)	36.1 (3.0)	3.6 (0.3)
Second treatment line	31.7 (2.6)	30.4 (2.5)	1.3 (0.1)
Salvage therapy lines	145.3 (12.1)	141.1 (11.8)	4.2 (0.3)
**Events**			
Opportunistic infections			
Bacterial	3.45%	3.57%	-0.12%
Fongal	5.58%	5.91%	-0.33%
Protozoal	1.92%	2.04%	-0.11%
Viral	1.48%	1.54%	-0.06%
Others	5.41%	5.47%	-0.06%
AIDS	72.08%	73.68%	-1.60%
AIDS free survival (years)	8.62	8.25	0.37
**INI resistance status, after first treatment line**			
No resistance	88.72%	55.84%	32.88%
**Mortality by cause**			
HIV/AIDS	21.98%	22.87%	-0.89%
HIV/AIDS including OIs	3.31%	3.47%	-0.16%
Other causes	78.02%	77.13%	0.89%
**Detailed costs (€2012–2014)**			
Cost of ART	€ 323,660	€ 315,506	€ 8,154
First line	€ 56,759	€ 51,690	€ 5,069
Subsequent lines	€ 266,901	€ 263,816	€ 3,085
Cost of care	€ 53,721	€ 54,583	-€ 862
Cost of death	€ 4,477	€ 4,618	-€ 141
Cost of tests	€ 4,344	€ 4,196	€ 148
Cost of OI	€ 2,493	€ 2,643	-€ 150
Cost of treatment switch	€ 1,306	€ 1,188	€ 118

AIDS: Acquired Immune Deficiency Syndrome; ART: AntiRetroviral Therapy; DTG: Dolutegravir; HIV: Human Immunodeficiency Virus; ICER: Incremental Cost-Effectiveness Ratio; INI: Integrase Inhibitor; LY: Life Year; OI: Opportunistic Infection; QALY: Quality Adjusted Life Years; RAL: Raltegravir

Overall, the model results show that DTG is more expensive than the strategy including RAL (€7,266 extra cost over the entire life of the patient) although the drug acquisition costs are the same for both agents. The higher costs associated with DTG are attributed to a higher life expectancy and consequently patients being treated longer (€8,154 cost of ART). In both arms, the cost of ART represents 83% of total costs, and the cost of care performed routinely accounts for approximately 14% of total costs in both groups, with a slightly lower cost to the DTG group linked to a higher CD4+ gain compared to RAL over time. As a result, the incremental cost-effectiveness ratio (ICER) for DTG is €21,048 per QALY gained compared to RAL.

Moreover, from the model results, patients treated with DTG present fewer opportunistic infections than patients treated with RAL and have an AIDS associated disease delayed by about four months. Consequently, fewer patients treated with DTG die from HIV/AIDS, or OIs than patients treated with RAL.

### Sensitivity analyses

**Fig 3:**
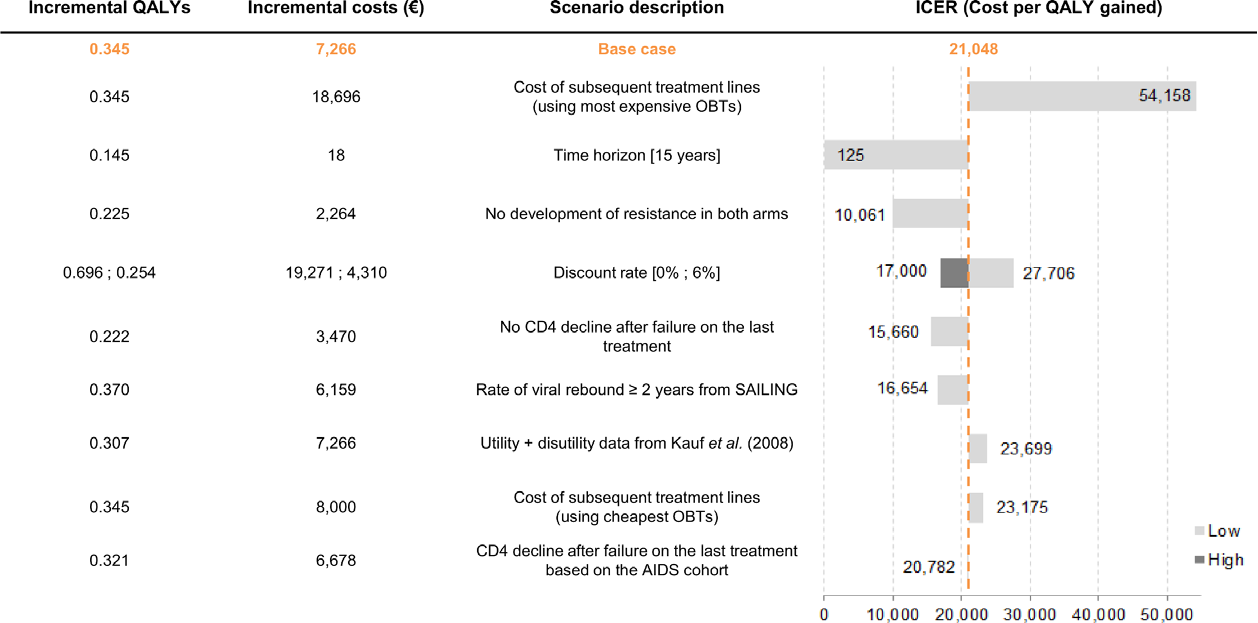
DSA—7 most influential parameters. AIDS: Acquired Immunodeficiency Syndrome; CD4: Cluster of Differentiation 4; DSA: Deterministic Sensitivity Analysis; ICER: Incremental Cost-Effectiveness Ratio; QALY: Quality Adjusted Life Year; OBT: Optimized Background Therapy.

The DSA results show that the ICERs DSA range from €125 to €54,158 per QALY for the seven most influencial parameters (Fig 3). The model results are most sensitive to the increase in subsequent treatment lines costs.

Sensitivity analyses show that, in the absence of discounting of costs and outcomes, there is a QALY and life year gain of 9.1 months and 8.3 months respectively for patients treated with DTG compared to RAL, highlighting the long-term benefits associated with this treatment. When the emergence of resistance to INI is not considered, the incremental costs decrease from €5,705 to €3,358 while the incremental QALYs decrease from 0.345 to 0.225, highlighting that the gain in QALYs (0.12) is directly attributable to the high genetic barrier to resistance of DTG.

Overall, the conclusion of the analysis showing the cost-effectiveness of DTG is robust to changes in most parameters (immunovirologic efficacy, utilities by CD4+ cell count level, discount rate, length of opportunistic infection, lack of emergence of resistance amongst others).

The results of analyses exploring price of DTG demonstrate that, at a 20% decrease in the reference price, DTG is dominant over the treatment strategy with RAL. At this price, DTG is less costly and provides a QALY gain. At a higher price of 20% of the price, the incremental cost/efficiency ratio is € 36,261.

## Discussion

The objective of the study was to assess the cost-effectiveness of DTG, in France, in TE and INI-naïve HIV adults patients with at least two ARV classes resistance, compared to RAL by adapting the previously published ARAMIS model.

The estimated incremental cost per QALY gain was €21,048 over a lifetime horizon. Patients treated with DTG lived longer and with a better quality of life (4.5 and 9.1 respectively discounted and undiscounted quality adjusted months) than patients with RAL. These benefits could be attributed to the better adherence to DTG associated with its convenient once-daily dosing compared to the twice-daily dosing of RAL. The DTG strategy was also more costly (additional cost of €7,266 compared to RAL). The increased cost was not due to a more costly drug, but to the long-term benefits of DTG in particular in terms of increased life expectancy, and therefore a longer time on treatment. The sensitivity analyses showed that the results were robusts, and few parameters had an impact on the ICER. Only the cost of subsequent therapy, with a maximum cost for OBTs was found to modify the conclusions of the analysis.

The average life expectancy of patients entering the model was estimated to be around 18 years. The reported life expectancy published in comparable models of ART varied greatly, between 8.9 and 29 years.[12, 36, 37] In the model, the QALYs accrued over a lifetime was estimated between 10.41 and 10.75. These numbers were consistent with recently published cost-effectiveness analyses for RAL, etravirine, or maraviroc, varying between 5.7 and 14.6 QALYs.[12, 36, 37] Several reasons could explain these differences, including varying stages of disease progression in considered populations, different sources of data for utilties, or the country of analysis. The impact of OIs in the model was negligeable with a low time spent in OI health states, reflecting the low incidence rates, in patients with controlled HIV.

The model had several limitations, due to challenges of establishing structural assumptions, parameter uncertainty, as well as model limitations.

First, treatment algorithms were difficult to establish, due to variations in practices, to the great number of treatments available, due to the choice of treatment depending on resistance and treatment history, and the heterogeneity within the profile of the SAILING trial patients. A scientific comittee was organised to define the treatment algorithm. The sensitivity analysis on the cost of treatment reflects the uncertainty around this algorithm, and its impact was found to be relatively important as an ICER of €54,158 was found for a maximum cost of subsequent therapies. However, this last hypothesis is not thought to be very probable as it is based on the assumption that all patients receive T-20 (Enfuvirtide). Although DTG has been used in the context of a NPP in France, in patients who have reached a therapeutic “dead end”, it was not considered as a possible option in the comparator’s treatment algorithm (RAL). It was assumed that the RAL strategy corresponded to a world without DTG, so as to ease the interpretation of results, and evaluate the impact of the introduction of DTG.

Efficacy data for DTG after one year were taken from clinical trials evaluating other treatments (RAL). This is thought to be a conservative assumption as DTG was shown to be superior to RAL at 48 weeks. However, the long-term treatment efficacy may have been over-estimated in the model compared to a real-life setting, as observed in another adaptation of the DTG ARAMIS model to Canada using real-life data.[38] The impact of this overestimation is however limited due to the inclusion of a threshold of 1,000 cells/m3 for CD4+ cell counts, and the limit set to the gain in CD4+ cell count in subsequent lines of treatment. Moreover, the efficacy data for subsequent lines of treatments are taken from published clinical trials and account for the number of active treatments. Sensitivity analyses were conducted to evaluate the impact of efficacy of subsequent lines and INI resistance on results. However, the conclusions of our analysis were not changed. The utility data were taken from the DTG clinical trials in naïve patients, rather than TE patients. These data were not coherent with the CD4+ cell counts categories, and different data was available in the literature. However, the sensitvity analysis did not show a large impact on results (€23,699 per QALY gained compared to the base-case). Furthermore, this analysis was based on a collective perspective, only accounting for direct costs.

Finally, it was not possible to conduct probabilistic sensitivity analyses due to running times. However, the DSA conducted to address the uncertainty around the points listed above showed that the conclusions of our analysis are robust.

## Conclusion

DTG is a cost-effective strategy compared to RAL in the management of TE INI-naive patients in France. Compared to RAL, DTG’s superior efficacy and higher barrier to resistance translated in TE patients staying longer on treatment, and a higher life expectancy. These results need to be confirmed with long-term real life data.
